# How to Obtain Forty Percent Less Environmental Impact by Healthy, Protein-Optimized Snacks for Older Adults

**DOI:** 10.3390/ijerph14121514

**Published:** 2017-12-06

**Authors:** Henrik Saxe, Signe Loftager Okkels, Jørgen Dejgård Jensen

**Affiliations:** 1 Department of Food Science, Section of Design and Consumer Behavior, Faculty of Science, Copenhagen University, DK-1958 Frederiksberg C, Denmark; 2 Dietetic and Nutritional Research Unit, Herlev and Gentofte University Hospital, DK-2820 Gentofte, Denmark; signe.loftager.okkels.01@regionh.dk; 3 Department of Food and Resource Economics, Section of Consumption, Bioethics and Governance, Faculty of Science, Copenhagen University, DK-1958 Frederiksberg C, Denmark; jorgen@ifro.ku.dk

**Keywords:** consequential life cycle assessment, global warming, monetized environmental impact, municipal kitchens, older adults, snack-meal recipes

## Abstract

It is well known that meals containing less meat are more sustainable, but little is known about snack-meals, which typically do not contain meat. This study investigates the diversity in environmental impacts associated with snack production based on 20 common recipes optimized for protein content, energy content and sensory aspects for older adults. The purpose is to improve sustainability of public procurement by serving more sustainable snack-meals. Public procurement serves Danish older adults over millions of snack-meals every year, and millions more are served in countries with a similar social service. The environmental impact of snack production was estimated by consequential life cycle assessment. The average impact of producing the 10 least environmentally harmful snacks was 40% less than the average impact of producing the 10 most harmful snacks. This is true whether the functional unit was mass, energy, or protein content, and whether the environmental impact was measured as global warming potential or the monetized value of 16 impact categories. We conclude that large-scale public procurement of snack-meals by private and municipal kitchens can be reduced by up to 40% if the kitchens evaluate the environmental impact of all their snacks and serve the better half more frequently.

## 1. Introduction

The environmental impact of food production and consumption is important to the local, regional and global environment [1]. Production of food and beverages is responsible for about half of the environmental impact caused by the personal activities of people living in industrialized countries [2], which in turn is responsible for about half of the overall environmental impact caused by these societies; the remaining half is caused by industry. Selection of healthy and environmentally friendly meals can reduce the environmental impact of food and beverage in the order of 25–65 percent [3,4]. This makes every meal, but particularly the numerous meals prepared in large numbers by central municipal or private kitchens, important, not only to the health of its customers but also to the overall environmental impact, both of which have significant socioeconomic implications [5]. 

While it is well known that the environmental impact of main meals largely depends on selecting meals with less meat, particularly beef [4,6,7], it is less clear whether selecting certain in-between meals above others can also contribute to reducing the environmental impact of food consumption, since snacks are typically meat-free. 

In Denmark, and similar countries with a high level of public service, older adults (older adults: EUROSTAT definition of people aged 65–84. http://ec.europa.eu/eurostat/statistics-explained/index.php/People_in_the_EU_%E2%80%93_statistics_on_an_ageing_society) who are unable to cook for themselves receive main meals and in-between meals produced by large municipal and private kitchens. For various reasons, older adults tend to eat inadequate amounts of food [8], and in-between meals (from here on called snacks) are therefore of particular importance to tempt the older adults to increase their energy and protein intake. The Danish recommendations for institution-diet advise people with poor appetite to eat at least three snack-meals daily [9]. In a study by Okkels et al. [10], 20 of the most popular/most ordered snacks were assessed by 30 older adults for flavor and appearance, and these snacks were further optimized in terms of energy, protein content and sensory aspects. The *primary objective* of this study is to determine if the variation in environmental impact of these 20 common snacks is significant and so large, that more often choosing snacks from the better half improves the overall sustainability. The *secondary objective* is to determine if there are certain ingredients in the snacks that are mainly responsible for their environmental impact. This is important since municipal and private commercial kitchens serve millions of snack-meals every year to older adults in Danish municipalities.

## 2. Materials and Methods

### 2.1. Recipes

**Figure 1:**
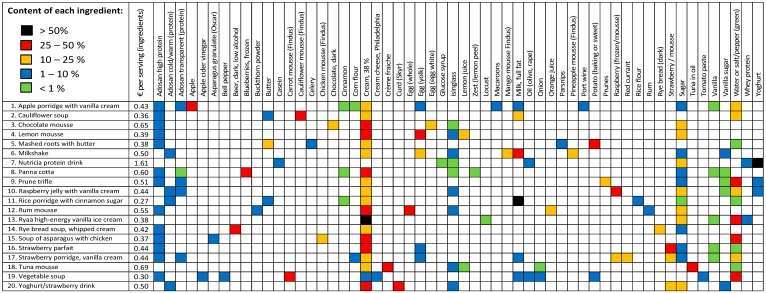
Ingredients in the 20 recipes of texture modified snacks where a specific amount of each ingredient is approximated due to proprietary rights. The relative content of all ingredients are given by the color codes. The estimated price of each snack is given in euros based on ingredients, but excluding the capacity cost.

Besides the importance of flavor and appearance, the nutritional criteria for all the optimized snacks were a content of at least 6 g protein and 400 KJ per 100 g, the typical weight of a single snack-meal. Figure 1 gives the approximated amounts of ingredients in the 20 recipes, while the precise recipes and energy consumption associated with production are given in Appendix A. 

### 2.2. Assessment of Environmental Responses and Monetized Impact

The environmental impact of the 20 recipes was calculated by consequential life cycle assessment (cLCA) of each snack recipe including all ingredients and cooking, baking, cooling and freezing. Life cycle assessment calculates the environmental impact of a product from soil to table using a range of impact categories and a selection of functional units. Consequential LCAs seek to identify the environmental consequences of a decision or a proposed change in a system under study (oriented towards the future), which means that market and economic implications of a decision are taken into account [11].

The scope of the present study included the response of 16 environmental impact categories associated with all activities, energy, and resource consumption from soil to produced and delivered snacks. These include human carcinogenic and non-carcinogenic toxicity (chloroethene-equivalents), respiratory inorganics (particulate matter with a diameter of ≤2.5 mm), ionizing radiation (Bq, the SI-derived unit of radioactivity, C_14_-eq), ozone layer depletion (chlorofluorocarbon 11), aquatic and terrestrial ecotoxicity (chloroethylene triethylene glycol-eq), nature occupation (agricultural land), global warming (CO_2_-eq), acidification (area unprotected ecosystems), aquatic (NO_3_-eq) and terrestrial (area unprotected ecosystems) eutrophication, respiratory organics (person·ppm^−1^·h^−1^), photochemical ozone effects on vegetation (m^2^·ppm^−1^·h^−1^), non-renewable energy (MJ primary), and mineral extraction (MJ extra).

However, for clarity, only data for the three most important impact categories (respiratory inorganics, nature occupation, and global warming) were presented separately in this study, along with the sum of the 13 other impacts. The environmental assessment was taken from the Ecoinvent database version 3.3 [12] using the Simapro 8.3 software (Simapro, Amersfoort, The Netherlands) [13]. The Stepwise 2006 version 1.05 method was applied to facilitate monetizing [14,15]. The Stepwise method combines methods from Impact 2002+ version 2.1 and EDIP 2003 with small modifications. Stepwise normalizes data by monetization expressed in Euro, thus calculating the potential socioeconomic cost of environmental externalities. All environmental impacts were calculated according to the ISO standard 14040 [16]. The functional units (i.e., references) were weight, energy content (MJ) or protein content in the manufactured snacks. The energy and protein contents of the ingredients were taken from the Danish FRIDA food database [17].

### 2.3. Statistics

A *t*-test was applied to determine the significance level of the difference between the 10 best and the 10 worst of the recipes in terms of global warming and monetized environmental. *T*-tests were also applied to determine if the 10 best recipes had a different content of the most impacting ingredients (cream and protein powder) than the 10 worst, and to determine if the 10 best recipes had a different cost of ingredients than the 10 worst. To investigate possible correlations between impact results using either of the two impact categories and any of the three functional units, a Spearman Rank-order correlation test was applied [18].

### 2.4. Cost of Ingredients

Based on recipes for the 20 optimized snack-meals, combined with food service prices for the ingredients, the ingredient costs per serving for the 20 snacks were calculated. Price data were for the period 2013–2014, supplied from one of the major Danish suppliers of groceries for food service operators.

For ingredients, where more than one variety was available from food service suppliers, the variety with the lowest price per kg was selected for the price calculation. This implies that the price estimates represent the lowest possible ingredient cost for the respective snacks. If further requirements to the ingredients are stated (e.g., that they should be organic as far as possible, should be domestically produced, should be semi-processed, etc.) the unit prices will tend to be higher.

The recipes for the 20 snacks include data on the amounts of ingredients and cooking, baking, cooling and freezing, but not full information about other energy use, personnel hours, depreciation and maintenance of kitchen facilities, waste management etc. Data from Danish municipal kitchens for provision of meals to the older adults suggest that ingredients on average constitute 30–40 percent of the total cost of meal service. This might suggest that the capacity costs would constitute around 1 € per snack, which should be added to the cost figures in Figure 1, although there is of course some variation in the time and energy requirements across snacks.

### 2.5. Packaging and Delivery

The snack-meals all weighed 100 g and were delivered on plastic trays for food with an average weight of 8 g covered with 0.5 g of plastic film. Small diesel vans, e.g., Fiat Ducato or Iveco were used to deliver meals from the kitchens to its customers in the shortest possible overall route.

## 3. Results

### 3.1. Recipes

Figure 1 shows four frequent commonalities of the 20 recipes: Fortification with protein powder for protein delivery, cream added for energy delivery and texture, sugar added for energy delivery and taste, and vanilla added for taste. Figure 1 also gives the estimated prices of the snacks. There are no significant differences between the average prices of snacks belonging to the best and worst half of global warming or monetized environmental impacts with any of the 3 functional units (kg, MJ or protein content). 

**Figure 2:**
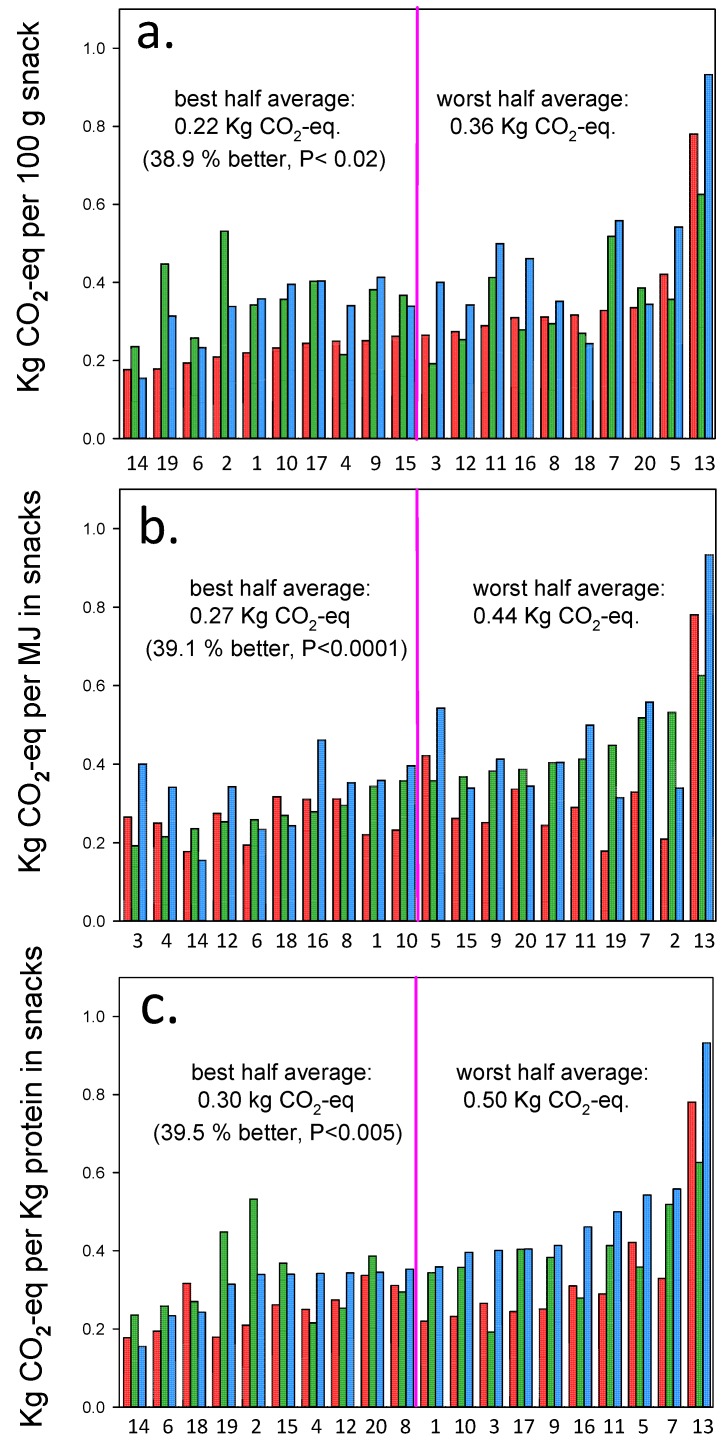
Global warming impact of 20 protein and energy fortified texture modified snacks. The numbers below each set of three bars refer to alphabetically ordered recipe numbers in Figure 1. The first bar (red) in each set indicates impact per 100 g snack, the second (green) impact per MJ energy content, and the third bar (blue) in each set indicates impact per kg protein in the snacks. Top figure (**a**) arranged by increasing impact relative to weight; middle figure (**b**) arranged by increasing energy content; bottom figure (**c**) arranged by increasing protein content. The average of the best and worst half in terms of climate impact is given in the figure together with the relative difference and associated significance level (*t*-test).

**Figure 3:**
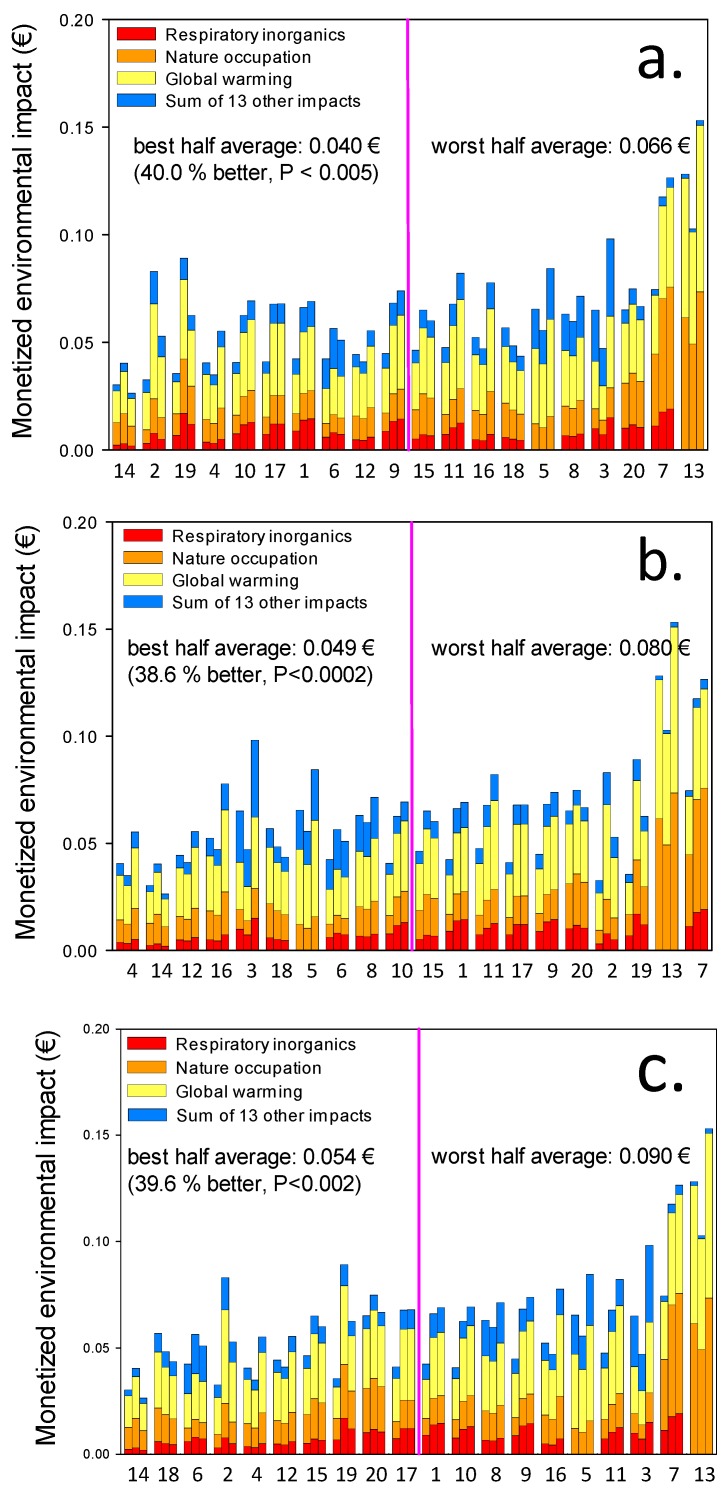
Monetized environmental impact of 20 protein and energy fortified texture modified snacks. Numbers below each set of three bars refer to alphabetically ordered recipe numbers in Figure 1. The first bar in each set of three bars indicates impact per 100 g snack, the second impact per MJ energy content, and the third bar in each set indicates impact per kg protein in the snacks. The colors indicate impact categories. Top figure (**a**) arranged by increasing overall impact relative to weight; middle figure (**b**) arranged by increasing overall impact relative to energy content; bottom figure (**c**) arranged by increasing overall impact relative to protein content. The average of the best and worst half in terms of monetized environmental impact is given in the figure together with the relative difference and associated significance level (*t*-test).

In spite of these commonalities, the global warming impact (Figure 2) and monetized overall environmental impact (Figure 3) of the 20 snacks differ significantly between the 10 best and 10 worst recipes (impacts, differences, and *p*-values are given in the figures).

### 3.2. Global Warming Impact

Figure 2 shows the global warming impact (GWP, kg CO_2_-eq) associated with production of the 20 snacks where the numbers below each sub-figure correspond to recipe numbers in Figure 1. Each set of three columns for each snack represents impact per 100 g (red), per MJ (green), and per kg protein (blue) respectively, arranged by increasing impact relative to snack weight (Figure 2a), relative to energy content (Figure 2b) and relative to kg protein (Figure 2c). On average, the best half of the snacks impacts global warming significantly about 40 percent less than the average of the worst half, whether the functional unit is weight, energy content (MJ) or protein content. However, the snacks belonging to the better half changes with the choice of functional unit. 

Only six snacks in Figure 2 are on the environmentally-friendly top-10 list for *both* energy and protein delivery: Rye bread soup with whipped cream, milkshake, lemon mousse, tuna mousse, rum mousse, and panna cotta. Only the first three of these are on the kg-based top-10 list of low GWP, demonstrating that environmental impact per kg (most commonly reported in the literature) is *useless* as a functional unit in relation to meaningful calculations of the GWP impact of snack-meals, i.e., in order to improve the energy and protein intake of older adults. The order of the global warming impact of the snacks differ between the three functional units, and the closest match is seen between weight and protein content (Figure 2). 

### 3.3. Monetized Overall Environmental Impact

Figure 3 shows the monetized environmental impact (€) measured over the 16 impact categories listed in Section 2.2 associated with the production of the 20 snacks. Each set of 3 bars for each snack represents the impact per 100 g (first bar), per MJ (middle bar) and per kg protein (right-hand bar) respectively, arranged by increasing impact relative to snack weight (top figure), energy content (middle figure) and kg protein (bottom figure).

The main monetized environmental impact is contributed by the GWP (yellow color) on average making up 46 percent of the overall impact. The impact of nature occupation (orange color), an analogy of biodiversity, makes up an average of 26 percent, respiratory inorganics 12 percent (red color), and the sum of the 13 other impact categories listed in Section 1.2 (blue color) on average make up only 16 percent of the monetized environmental impact of the snacks. These percentages vary among the snacks (Figure 3).

On average, the best half of the snacks have a monetized environmental impact which is significantly about 40 percent less than the average of the worst half, whether the functional unit is weight, energy content (MJ) or protein content (Figure 3). However, the snacks included in the better half vary with the choice of functional unit.

Only five snacks are on the top-10 list for *both* energy and protein delivery in Figure 3: Rye bread soup with whipped cream, milkshake, lemon mousse, tuna mousse, and rum mousse. Since only four of these are also on the kg-based top-10 list of low overall environmental impact and the ranking differ among all three functional units, the environmental impact per kg is *useless* as a functional unit in relation to meaningful calculations of the GWP impact of food. The order of the global warming impact of the snacks differ between the three functional units.

### 3.4. Correlation between Indicators

Spearman-rank-order correlations between the impacts measured by the three functional units (kg, MJ, or protein content) combined with the two investigated impact categories (GWP or €) are significant (*p* < 0.04 to *p* < 0.00003) in 11 of the 15 combinations. In spite of this, Figure 2 and Figure 3 clearly demonstrate that the choice of functional unit and impact category are important to select snacks according to the specific purpose, i.e., to deliver energy and protein to the older adults.

### 3.5. What Makes a Snack Less Sustainable?

Table 1 illustrates the 4 to 6 most environmentally harmful ingredients in each of the 20 snacks totalling nearly 100% of the total impact. When comparing these impacts in the best and worst recipes, Table 1 shows that cream is the first or second most impacting ingredient measured both as GWP and as monetized overall environmental impact in 18 of the 20 recipes. Cream is not an ingredient in Nutricia^®^ protein drink, and in prune trifle cream takes second place only when the impact is measured as GWP and third place when the impact is measured by the monetized overall environmental impact. 

In the 10 recipes with the lowest GWP impact relative to energy-content (Figure 2b), the average cream content is 44%, and in those with the highest GWP impact it is 27%. A *t*-test reveals that the 38% lower cream content in the 10 snacks with the highest GWP impact compared with the 10 snacks with the lowest impact is significant (*p* < 0.02). Similar results are found for GWP impact relative to protein-content, monetized overall environmental impact relative to MJ and to protein content, but the results are less or not significant, respectively 34% lower (*p* < 0.03), 31% lower (*p* < 0.05), and 16% lower cream content in the 10 snacks with the highest impact compared with the 10 snacks with the lowest impact (*p* < 0.23).

Table 1 shows that the protein powders Adosan^®^ (a registered trademark including four types, *all* sourced from veal and milk: “cold/warm”, “high-energy”, “high protein” and “transparent”), *whey and casein are on average the second most impacting ingredients measured both as GWP (CO*_2_*-eq) and as monetized overall environmental impact (€) in 18 of the 20 recipes*. The exact composition of Adosan^®^, which cannot be publicly disclosed, is known to the authors and used in the LCA calculations. In rum mousse, eggs supply the protein, and in *t*una mousse canned tuna supplies the protein. In the 10 recipes with the lowest GWP impact relative to protein-content (Figure 2c), the average protein powder content is 12%, and in those with the highest GWP impact it is 31%. A *t*-test reveals the 163% higher protein powder content in the 10 snacks with the highest GWP impact compared with the 10 snacks with the lowest impact is significant (*p* < 0.02). Similar results are found for GWP impact relative to energy-content (Figure 2b), monetized overall environmental impact relative to MJ (Figure 3b) and to protein content (Figure 3c), but the results are less or not significant, respectively 59% higher (*p* < 0.14), 128% higher (*p* < 0.04), and 109% higher protein powder content in the 10 snacks with the highest impact compared with the 10 snacks with the lowest impact (*p* < 0.06).

### 3.6. Packaging and Delivery 

Using 8 g plastic trays and 0.5 g plastic film for cover, the environmental impact associated with packaging added a global warming impact of 0.02 kg CO_2_-eq and a monetized overall environmental impact of 0.002 € per meal. This makes up only 2–10 percent of the production impact, which does not affect the conclusion that it is the production rather than packaging that impacts the environment.

The delivery vans typically travelled about 1 km for delivery to each customer. Deliveries consist of a single main meal, and up to seven main meals in addition to the snack-meals. The environmental impact of meal delivery by medium size vans ranged from 0.026 kg CO_2_-eq and 0.005 € per meal, and up to 0.435 kg CO_2_-eq and 0.09 € per meal depending on the average distance between customers (0.92–1.56 km) and the frequency of meal delivery (1–7 times per week). If the meals are delivered on a weekly basis, the environmental impact of delivery is insignificant compared with the meal production. A minor importance of packaging and delivery was also found by [7].

## 4. Discussion

### 4.1. A Path towards Greater Sustainability

Snack meals often account for half the energy intake of older adults in Denmark. If further tests of the snack recipes discussed in this paper are successful in terms of liking, life quality, physical performance, cognitive status and body weight conservation of the older adults, the recipes could be produced and offered to this group on a larger scale. Approximately 114,000 people received snack meals in Denmark in 2016 from private and municipal kitchens. With two snack meals per day, this totals 83 million snack meals per year. 

With a potential of 40 percent savings on global warming by choosing the best half of the snack-meals for the older adults in Denmark, it would equal an annual saving of 1165 t CO_2_, or the annual emission from driving a Euro 5 passenger car approximately 10 million km. Even with the environmentally best half of the snack-meals being chosen more often than the worst half, there can be substantial environmental savings, with a reasonably broad choice of recipes. If you consider the potential savings on a larger European scale, the savings on environmental impact grows far bigger. 

### 4.2. Choice of Functional Unit and Impact Category

Which functional unit and impact category is the best to point the kitchens towards snacks with the lowest environmental impact? The answer to the first question depends on the *purpose* of the snack-meals, whether they are meant to improve the energy or protein content of the daily food intake of the older adults, or in this study both. There is increasing focus on the choice of functional unit when LCAs are used to compare foods [19,20]. Recently, Ref. [21] pointed out that protein quality may be an even better functional unit that protein quantity. The most common functional unit in LCA studies, weight (kg), was included in this study to be able to calculate the others, but it proved meaningless in selecting the most sustainable snack-meals, with the purpose of delivering *both* energy and proteins to the older adults.

The GWP is the dominant environmental impact of snack production, but it makes up a little less than half of the average monetized overall environmental impact of all snacks. So the answer to the second question is that the sum of the monetized impacts of all 16 impact categories is the best environmental indicator, since it covers the overall environmental impact better than any single category, even the GWP. Therefore, even though the use of different functional units and impact categories was shown to be significantly correlated in 11 out of 15 combinations, the choice of functional unit and impact category matters as it affects the ranking of recipes in terms of environmental sustainability. 

### 4.3. Which Ingredients Mainly Cause the Environmental Impact? 

All ingredients cause environmental impact, but as Table 1 shows, it is particularly cream and protein powders that are responsible for the environmental impact of the 20 different snacks. The 10 snacks with the highest impact on average (surprisingly) contain the least cream, and (not surprisingly) the most protein powder. Does this indicate that a generic increase in cream and decrease in protein powder content would increase sustainability of the snacks? No, an increase in cream would decrease sustainability in any given snack and add unnecessary energy, while a decrease in protein powder content would indeed increase sustainability, but miss the purpose of the snacks to supplement older adults with more protein in their daily food consumption. Reducing protein powder and increasing cream content in the recipes may not even be compatible with healthy and tasty snacks. There is therefore no *general* advice on how to modify the recipes for all snacks in order to reduce their environmental impact. 

The logical way forward is to more frequently choose snacks among the 10 least impacting recipes and less frequently choosing snacks among the 10 with the highest environmental impact in order to reduce the environmental impact of the snacks offered to the older adults by up to 40 percent. Each private and municipal kitchen should evaluate the environmental impact of all their snacks, and serve the better half more frequently.

### 4.4. Prices Are Not Correlated with Environmental Impact

Although there is a wide range of snack prices (0.27 € to 1.61 € per serving, or a cheapest-to-most-expensive ratio of 6.2) based on the cost of ingredients and cooking, baking, cooling and freezing, there is no economic incentive not to choose *the best half* of the snacks in terms of either climate change impact or the monetized overall environmental impact. The average cost of all snacks is about 0.5 €, and the average price of the five snacks that are on the top-10 list for both energy and protein delivery in Figure 3 is similarly about 0.5 €. 

The *total* cost of any snack also include the capacity cost, and this was estimated as twice (around 1 €) the average cost of ingredients. With more information on energy use, personnel hours, depreciation and maintenance of kitchen facilities and waste management that was available for this study, this value could be more precisely calculated. Adding the fabricating cost to that of the ingredients reduces the relative cost difference between individual snack-meals (1.27 € to 2.61 €) reducing the cheapest-to-most-expensive ratio to a third: 2.1.

We recommend that the choice of snacks should be selected based on *taste/acceptability* (it is important that the older adults eat the additional snack-meals), *health* (older adults are vulnerable), and *environmental impact* (food is a major contributor to our overall environmental impact)—in that order—but without neglecting *any* of these three aspects of snack-meals. Fortunately, low environmental impact often follows a positive health impact of foods [22], and monetized health impact has been shown to be more important than the monetized environmental impact [4,5]. The price of the snack should not influence the choice of snacks to be served to the older adults.

## 5. Conclusions

There is a 40 percent improvement in environmental impact of snack-meals for the older adults if choosing the environmentally best half rather than the worst half of 20 snack-meals, independent of the functional unit (weight, energy or protein content) and impact category (GWP or the monetized value of 16 impact categories). Choosing more snacks from the best half improves the overall sustainability. However, taste, health and environmental impact should, in that order, decide the choice of snack-meals for the older adults. The data provided by this study give no support for general modification of the recipes. However, future studies should investigate whether protein powders sourced from plants rather than from animals could result in a lower environmental impact of snack meal recipes with added protein powder.

In view of the large differences between the least and the most environmentally harmful snacks, it is advised that public and private providers of meals for the older adults (and other population groups) make an environmental inventory of all their meals, in order to offer not only the tastiest, healthiest and most attractive, but also the environmentally best snack meals. A conscious diet choice is now a proven path towards greater sustainability.

## Figures and Tables

**Table 1:** Ingredients of the 20 snack meal recipes and their relative share of total impact in terms of GWP (climate) and monetized overall environmental impact (€). 97–100% of the total impact is accounted for by 3–6 ingredients and processes. Adosan stands for one of several types of Adosan^®^ protein powders.

Recipe	Type of Impact	Ingredients and Their Share of Total Impact in Terms of GWP (Climate) and Monetized Overall environmental Impact (€)						Total Impact
1. Apple porridge with vanilla cream	GWP	Adosan 39%	cream 22%	apples 14%	port + macaroons 9%	vanilla 7%	milk 5%	97%
	€	Adosan 31%	cream 22%	apples 17%	port + macaroons 14%	vanilla 8%	milk 7%	98%
2. Cauliflower soup	GWP	butter 29%	cream 27%	cauliflower 18%	milk 15%	Adosan 10%	-	99%
	€	butter 25%	cream 32%	cauliflower 19%	milk 16%	Adosan 7%	-	99%
3. Chocolate mousse	GWP	cream 49%	chocolate 31%	egg white 12%	Adosan 3%	sugar 2%	-	98%
	€	chocolate 54%	cream 37%	egg white 5%	Adosan 1%	sugar 1%	-	99%
4. Lemon mousse	GWP	cream 62%	eggs 22%	lemon 5%	Adosan 5%	boiling 2%	sugar 3%	98%
	€	cream 71%	eggs 15%	lemon 8%	Adosan 5%	boiling 1%	sugar 0.2%	100%
5. Mashed roots with butter	GWP	butter 69%	cream 19%	Adosan 7%	potatoes 5%	-	-	99%
	€	butter 62%	cream 24%	potatoes 9%	Adosan 5%	-	-	99%
6. Milkshake	GWP	milk 27%	cream 26%	Adosan 25%	fruits 12%	vanilla 5%	egg yolk 4%	100%
	€	milk 20%	cream 22%	Adosan 15%	cane sugar 25%	fruits 8%	vanilla 7%	98%
7. Nutricia protein drink	GWP	casein 42%	milk 35%	whey powder 12%	sunflower oil 9%	Sugar 1%	-	99%
	€	milk 35%	casein 30%	whey powder 8%	sunflower oil 24%	Sugar 0%	-	98%
8. Panna cotta	GWP	cream 64%	Adosan 17%	blueberries 13%	vanilla 3%	boiling 1%	sugar 0.5%	98%
	€	cream 58%	Adosan 11%	blueberries 12%	sugar 13%	vanilla 3%	boiling 1%	98%
9. Prune trifle	GWP	Adosan 46%	cream 21%	prunes 21%	yoghurt 8%	vanilla 2%	sugar 1%	98%
	€	Adosan 40%	prunes 23%	cream 22%	yoghurt 10%	vanilla 3%	boiling 1%	100%
10. Raspberry jelly with vanilla cream	GWP	Adosan 44%	cream 25%	raspberries 17%	yoghurt 9%	isinglass 3%	vanilla + sugar 2%	99%
	€	Adosan 40%	cream 26%	raspberries 18%	yoghurt 11%	isinglass 2%	vanilla + sugar 2%	99%
11. Rice porridge with cinnamon sugar	GWP	milk 41%	cream 19%	butter 14%	rice 8%	Adosan 7%	cinnamon 7%	97%
	€	milk 41%	cream 21%	butter 11%	cinnamon 10%	rice 9%	Adosan 6%	98%
12. Rum mousse	GWP	cream 51%	eggs 28%	rum 8%	buckthorn 4%	orange 3%	isinglass 2%	99%
	€	cream 59%	eggs 19%	rum 7%	buckthorn 6%	orange 6%	isinglass 1%	100%
13. Ryaa high-energy vanilla ice cream	GWP	whey protein 62%	cream 34%	freezing 4%	-	-	-	99%
	€	cream 58%	whey protein 39%	freezing 3%	-	-	-	100%
14. Rye bread soup, whipped cream	GWP	cream 40%	Adosan 26%	rye bread 18%	beer 12%	boiling 4%	sugar 1%	100%
	€	cream 43%	Adosan 16%	rye bread 24%	beer 14%	boiling 3%	-	100%
15. Soup of asparagus with chicken	GWP	cream 55%	chicken 22%	asparagus 10%	Adosan 10%	boiling 3%	-	100%
	€	cream 57%	chicken 25%	asparagus 10%	Adosan 6%	boiling 2%	-	100%
16. Strawberry parfait	GWP	cream 67%	freezing 11%	strawberry 10%	Adosan 6%	vanilla 3%	egg yolk 2%	99%
	€	cream 74%	freezing 6%	strawberry 11%	Adosan 4%	vanilla 3%	egg yolk 1%	100%
17. Strawberry porridge, vanilla cream	GWP	Adosan 42%	cream 25%	berries 15%	vanilla 7%	milk 7%	boiling 2%	98%
	€	Adosan 39%	cream 28%	berries 15%	vanilla 9%	milk 6%	boiling 1%	98%
18. Tuna mousse	GWP	sour cream 49%	cream 30%	tuna 19%	isinglass 1%	-	-	99%
	€	sour cream 51%	cream 31%	tuna 17%	isinglass 1%	-	-	99%
19. Vegetable soup	GWP	vegetables 36%	cream + cheese 29%	Adosan 11%	milk 9%	oil + vinegar 8%	boiling 4%	98%
	€	vegetables 34%	cream + cheese 28%	oil + vinegar 21%	milk 7%	Adosan 6%	boiling 2%	98%
20. Yoghurt/strawberry drink	GWP	cream 40%	curd 34%	Adosan 14%	strawberry 7%	vanilla 2%	isinglass 2%	99%
	€	curd 41%	cream 38%	Adosan 9%	strawberry 6%	vanilla 4%	Isinglass 1%	100%

## Appendix A. Optimized Nutrient Rich Recipes of Snacks for Older Adults

These studied recipes were developed by Signe Okkels. In a first phase, they were evaluated by 20 older adults at three nursing homes on Zealand, Denmark. Based on their feedback regarding taste and appearance, the recipes were optimized and their nutrient content adjusted by a dietician *to ensure that are suitably balanced in essential nutrients with focus on energy and protein content.* Snacks based on the resulting recipes below are expected to be served for older adults in several Danish municipalities. Each portion equals 100 g.

**Recipe 1** Apple porridge with vanilla cream

Ca. 40 portions = 4083 g

Apple porridge:

125 g Adosan^®^ ‘transparent’ (www.adosan.dk)

1935 kg frozen apple slices

500 mL water

2 cinnamon pods = 2 × 2.5 g = 5 g

Energy for freezing and boiling

Enriched vanilla cream:

288 g organic whole milk

69 g Egg Yolks (Cater^®^)

87 g organic sugar

24 g Maizena^®^ (maize flour)

5 vanilla pods = 5 × 5 g = 25 g

462 g cream (38%)

3 g salt

150 g Adosan^®^ ‘high protein’

310 g small macaroons (Karen Wolf^®^)

100 g port

**Procedure:** Apple porridge: Steam the frozen apple slices until tender, together with the cinnamon in a pot with a lid (ca. 15 min). Let it cool down. Vanilla cream: Milk, yolks, sugar, Maizena, salt and vanilla powder from pods are blended, boiled and left to cool off. Whip the cream lightly with Adosan^®^. Carefully the vanilla cream and whipped cream together to produce the vanilla cream. Crush the macaroons in a plastic bag to form a fine powder, and add port. Portions consist of 30 g apple porridge, 15 g macaroon powder, another 30 g apple porridge and 25 g vanilla cream on top.

**Nutrients per 100 g:** 623 KJ, 6 g protein, 5.2 g fat, 18 g carbohydrates.

**Recipe 2** Cauliflower soup

Ca. 23 portions = 2313 g

1000 g cauliflower mousse (Findus^®^)

400 g water

100 g butter

400 whole milk

300 g cream (18%)

100 g Adosan^®^ ‘high protein’

10 g salt

3 g pepper

Energy for boiling

**Procedure:** Mix all ingredients and boil briefly.

**Nutrients per 100 g:** 402 KJ, protein 6.1 g, fat 6.6 g, 2.9 g carbohydrates.

**Recipe 3** Chocolate mousse

Ca. 26 portions = 2666 g

4 leaves of isinglass (4 × 4 g = 16 g)

600 g egg whites

600 g dark chocolate (70% cocoa)

600 g sugar

800 g cream (38%)

50 g Adosan^®^ ‘high protein’

Energy for heating and cold storage of the chocolate mousse

**Procedure:** Whip sugar and egg whites until stiff. Carefully melt the chocolate by heating water around the bowl with chocolate. Pour the chocolate in the egg-sugar mixture. Whip the cream and carefully mix it with the chocolate-sugar-egg-mixture.

**Nutrients per 100 g:** 1422 KJ, 6.2 g protein, 18 g fat, 37.6 g carbohydrates.

**Recipe 4** Lemon mousse

Ca. 39 portions = 3940 g

40 g isinglass

1000 g egg

960 g sugar

440 g freshly squeezed lemon juice

1400 g cream (38%)

100 g Adosan^®^ ‘high protein’

Energy for heating and cold storage of the lemon mousse

**Procedure:** Whip a whole egg with sugar for a thick eggnog in a large pot, possibly over 2–3 batches with max. 6 eggs at a time. Whip the cream until stiff and add Adosan^®^.

Hydrate the isinglass in cold water for 10 min, squeeze out excess water, heat and then cool to below 4 °C before adding it gradually to the eggnog through a sieve while stirring. Carefully add whipped cream, while the mousse is complete by adding lemon juice until the taste is right.

**Nutrients per 100 g:** 1171 kJ, 7.4 g protein, 16 g fat, 26.6 g carbohydrates.

**Recipe 5** Mashed roots with butter

12 portions = 1201 g

250 g butter

75 g Adosan^®^ ‘high protein’

6 g salt

90 g sweat potato

40 g organic celery (root)

220 g organic cream (38%)

470 g baking potatoes

50 g parsnips

Energy for boiling

**Procedure:** Peel and chop the roots. Melt the butter together with the chopped roots. Add Adosan^®^ and cream. Boil the roots slowly and blend it all.

**Nutrients per 100 g:** 1198 KJ, 7.9 g protein, 24.2 g fat, 9.5 g carbohydrates.

**Recipe 6** Milkshake

17 portions = 1700 g

250 g pineapple puree (Findus^®^)

250 g mango puree (Findus^®^)

500 g whole milk

200 g cream (38%)

200 g pasteurized egg yolks

100 g sugar

150 g Adosan^®^ ‘warm/cold’

Ca. 2 table spoons vanilla sugar = 2 × 10 g = 20 g

Ca. 2 table spoons of lemon juice = 30 g

Energy for blending

**Procedure:** Blend all ingredients for at least 90 s.

**Nutrients per 100 g:** 734 KJ, 8.1 g protein, 9.6 g fat, 13.5 g carbohydrates.

**Recipe 7** Nutricia^®^ protein drink, yoghurt style. Vanilla-lemon taste (factory-produced) http://nutricia.dk/images/uploads/pdf/Datasheet_Drinks_2015/Nutridrink_Yoghurt_style.pdf

1 portion = 100 g

0.4 g glucose syrup (specified on label)

10.5 g saccharose (specified on label)

4.4 g whey powder (specified on label)

2.5 g Casein (specified on label)

82.5 g skim milk yoghurt (guestimate)

5.7 g rape/sunflower oil (guestimate)

0.5 g pectin (guestimate)

Energy for production (guestimate)

**Procedure:** Bought from factory.

**Nutrients per 100 g:** 630 kJ, 5.9 g protein, 5.8 g fat, 18.7 g carbohydrates.

**Recipe 8** Panna cotta

33 portions = 3297 g (1902 g panna cotta + 1395 g fruit puree)

Panna cotta

1500 g cream (38%)

135 g powdered sugar

12 g isinglass

15 g vanilla sugar

15 g organic lemon peel

225 g Adosan^®^ ‘high protein’

Energy for cooling

Fruit puree

1200 g frozen blueberries

150 g cane sugar

1.5 pod vanilla = 7.5 g vanilla pod

3 cinnamon pods = 3 × 2.5 g = 7.5 g cinnamon pod

30 g Adosan^®^ ‘transparent’

Energy for freezing and boiling

**Procedure:** Panna cotta: Hydrate the isinglass. Heat the cream to boiling with the other ingredients, while stirring carefully. Melt the isinglass in the lukewarm creamy substance. Place portions under cooling.

Fruit puree: Boil berries, sugar, Adosan^®^, cinnamon and vanilla pods. Cool, and remove the pods before mixing with a hand blender. Run the produce through a sieve and put it on top of the Panna Cotta.

**Nutrients per 100 g:** 1056 kJ, protein 8.7 g, 17.7 g fat, 14.6 g carbohydrates.

**Recipe 9** Prune trifle

33 portions = 3300 g

Prune trifle

600 g prunes

1500 g water

170 g sugar

10 g vanilla sugar

150 g Adosan^®^ ‘transparent’

Energy for boiling

Cream

400 g cream (38%)

300 g plain yoghurt, 3.6% fat

60 g sugar

100 g Adosan^®^ ‘high protein’

10 g vanilla sugar

**Procedure:** Prune porridge: Hydrate the prunes in water for 24 h. Then boil the prunes with sugar, vanilla and Adosan^®^. Let the porridge cool down. Cream: Whip the cream and mix the cream ingredients into the cream, and place the cream on top of each portion prune trifle.

**Nutrients per 100 g:** 651 KJ, 6 g protein, 5.1 g fat, 20.6 g carbohydrates.

**Recipe 10** Raspberry jelly with vanilla cream

Ca. 31 portions = 3158 g (2268 g jelly + 890 g cream)

Raspberry jelly

1200 g raspberry puree

300 g sugar

120 g Adosan^®^ ‘transparent’

48 g isinglass

600 g water

Energy for cooling

Cream

400 mL = 420 g cream (38%)

300 g plain yoghurt (3.6% fat)

60 g sugar

100 g Adosan^®^ ‘cold/warm’

10 g vanilla sugar

Energy for cooling

**Procedure:** Raspberry jelly: Boil water and sugar with Adosan^®^. Melt hydrated isinglass into the substance. Add the raspberry puree and stir. Pour into small bowls or cups and preserve cold. Cream: Whip the cream; add sugar, vanilla sugar and Adosan^®^. Stir with yoghurt and put it all over the jelly, when this is solid.

**Nutrients per 100 g:** 609 KJ, 7.2 g protein, 5.7 g fat, 17.1 g carbohydrates.

**Recipe 11** Rice porridge with cinnamon sugar

Ca. 25 portions = 2550 g (2375 g porridge + 130 g butter + 32.5 g sugar + 32.5 g cinnamon)

25 × 3 g = 75 g butter

25 × 1 g sugar = 25 g sugar

25 × 1 g cinnamon = 25 g cinnamon

315 g cream (38%)

1.7 L whole milk

250 g rice flour

2 table spoon salt = 2 × 5 g = 10 g salt

100 g Adosan^®^ ‘cold/warm’

Energy for boiling

**Procedure:** The porridge is stirred and boiled under lid at low heat for approx. 50 min. Add salt for taste. The core temperature must be 80 °C. Serve the porridge with cinnamon sugar and butter.

**Nutrients per 100 g** (including butter and cinnamon sugar)**:** 712 KJ, 5.6 g protein, 10.1 g fat, 14 g carbohydrates.

**Recipe 12** Rum mousse

31 portions = 3100 g

1000 g whole eggs

400 g powdered sugar

40 g Isinglass

150 g rum

1000 g cream (38%)

400 g orange juice

50 g Adosan^®^ ‘high protein’

60 g buckthorn powder (dried berries, blended, strained and dried to form the powder).

Energy for water bath

**Procedure:** Whip the whole eggs with powdered sugar for a thick eggnog. Place the isinglass in an excess of cold water for at least five minutes. Melt the hydrated isinglass in a water bath. Whip the cream. Let the isinglass cool off with rum and juice (careful not to add too much rum). Slowly pour the isinglass in the eggnog while stirring. Add the whipped cream carefully.

**Nutrients per 100 g:** 1020 KJ, 7.6 g protein, fat 15.5 g, 15.4 g carbohydrates.

**Recipe 13** Ryaa high-energy vanilla ice cream (Ryaa dairy, www.Ryaais.dk/energitaet-is)

100 portions = 10,000 g

6000 g cream (38%)

30 g vanilla

1200 g sugar

600 g whey protein

39 g locust bean-seed flour

2131 g water

Energy for the freezing

**Procedure:** Bought from factory.

**Nutrients per 100 g:** 1292 kJ, 6.69 g protein, 14 g carbohydrates, 24.44 g fat.

**Recipe 14** Rye bread soup, whipped cream

Ca. 15 portions = 1550 g

350 g dark rye bread

750 g alcohol-free beer

50 g brown sugar

150 g Adosan^®^ ‘high protein’

250 g cream (38%)

Energy for boiling the soup and whipping the cream

**Procedure:** Hydrate the rye bread in the beer for half an hour. Add brown sugar and Adosan^®^. Blend the soup and boil it at medium heat for ca. 5 min while stirring.

**Nutrients per 100 g**: 747 kJ, 11.4 g protein, 6.4 g fat, 16.5 g carbohydrates.

**Recipe 15** Soup of asparagus with chicken

Ca. 18 portions = 1827 g

815 g water from canned asparagus slices

112 g Oscar^®^ asparagus soup granulate

For sustainability LCA we used the information on the contents of 3 kg granulate contains: 1430 MJ, 3.5 g fat, 69 g carbohydrates, 7.5 g protein, 0 g fiber and 9.99 g salt (NaCl). Since Oscar^®^ and Nestle^®^ keep the ingredients list of their products confidential, the nutrient information and label declaration was used to guestimate the ratio of ingredients, i.e., 5% asparagus extract + 1440 g skim milk powder + 250 g modified starch + 27 g palm oil + 380 g sugar + an insignificant amount of maltodextrin + 3.33 g salt, and insignificant amounts of aroma, locust bean flour, guar gum, and spice extracts.

200 g chicken puree (Findus^®^)

600 g cream (38%)

100 g Adosan^®^ ‘high protein’

Energy for boiling

**Procedure:** Mix all ingredients and boil. Add salt for taste.

**Nutrients per 100 g:** 716 KJ, 7 g protein, 7.7 g, Fat 13.2 g, 5.7 g carbohydrates.

**Recipe 16** Strawberry parfait

Ca. 20 portions = 1990 g

570 g strawberry puree (frozen, Ardo^®^)

1.5 vanilla pods = 1.5 × 5 g = 7.5 g

189 g sugar

189 g egg yolks

951 g cream (38%)

3 g salt

80 g Adosan^®^ ‘high protein’

Energy for freezing

**Procedure:** Mix thawed, but cool fruit puree with the other ingredients in a mixing bowl. Carefully add the whipped cream to this, fill it in plastic cups, and freeze.

**Nutrients per 100 g:** 1099 KJ, 6.5 g protein, 20.5 g fat, 13.2 g carbohydrates.

**Recipe 17** Strawberry porridge, vanilla cream

33 portions = 3245 g

Porridge

363 g organic strawberries (frozen)

363 g red currant (frozen)

363 g raspberries (frozen)

120 g sugar

48 g Tapioca Maizena^®^

130 Adosan^®^ ‘transparent’

800 g water

Energy for boiling

Enriched vanilla cream

288 g organic whole milk

69 g egg yolks (Cater^®^)

87 g organic sugar

24 g Maizena^®^ (maize flour)

5 vanilla pods = 5 × 5 g = 25 g

462 g cream (38%)

3 g salt

100 g Adosan^®^ ‘high protein’

Energy for boiling

**Procedure:** Boil the berries, sugar in the majority of the water and add Maizena^®^ dissolved in a small amount of water. Cool down.

**Nutrients per 100 g:** 599 kJ, 6 g protein, 6.6 g fat, 14.2 g carbohydrates.

**Recipe 18** Tuna mousse

7 portions = 700 g

8 g isinglass

150 g cream (38%)

250 g crème fraiche (38%)

2 g chopped onion

4 g salt

1 g pepper

280 g tuna in oil (http://www.johnwest.dk/)

5 g lemon juice

Energy for refrigeration

**Procedure:** Pour the tuna onto a sieve to relieve it of water. Blend the tuna, onion, sour cream, and lemon juice. Hydrate four leaves of isinglass in cold water, wring it for water, mix it with a couple of spoonful’s boiling water, and pour it slowly into the mixture while stirring. Whip the cream and add it to the mixture. Add salt, pepper and lemon juice for taste. Place the mousse in a refrigerator to facilitate drawing patterns in the surface. Place in a refrigerator for a minimum of 3 h.

**Nutrients per 100 g:** 1201 KJ, 12.2 g protein, 26 g fat, 1.8 g carbohydrates.

**Recipe 19** Vegetable soup

Ca. 23 portions = 2292 g

50 g olive oil

600 g carrot puree (Findus^®^)

150 g baking potatoes

50 g chopped, frozen onion

50 g red bell pepper

100 g tomato puree (Ponte Sisto^®^, organic; http://psimport.dk/produkter/tomatkoncentrat)

600 g water

200 g whole milk

200 g cream (38%)

100 g Adosan^®^ ‘high protein’

120 g Philadelphia cream cheese

3 tablespoons of apple cider vinegar = 45 g

6 teaspoons of salt = 24 g

1 teaspoon of pepper = 3 g

Energy for boiling

**Procedure:** Fry the bell peppers in the oil and add all ingredients. Bring to boil, and blend the soup the wanted fluidity. Add salt and pepper for taste.

**Nutrients per 100 g:** 441 KJ, 5.8 g protein, 7.2 g fat, 4 g carbohydrates.

**Recipe 20** Yoghurt/strawberry drink

Ca. 15 portions = 1453 g

400 g curd (‘skyr’)

450 g cream (38%)

300 g strawberry puree (Findus^®^)

150 g sugar

120 g Adosan^®^ ‘cold/warm’

18 g isinglass

15 g vanilla sugar

**Procedure:** Hydrate the isinglass and melt it. Stir the other ingredients together, and slowly add the melted isinglass. Put the drink in small bowls or cups.

**Nutrients per 100 g:** 928 kJ, 9.5 g protein, 12.9 g fat, 16. 4 g carbohydrates.
